# A Prognostic Model for Estimating the Time to Virologic Failure in HIV-1 Infected Patients Undergoing a New Combination Antiretroviral Therapy Regimen

**DOI:** 10.1186/1472-6947-11-40

**Published:** 2011-06-14

**Authors:** Mattia CF Prosperi, Simona Di Giambenedetto, Iuri Fanti, Genny Meini, Bianca Bruzzone, Annapaola Callegaro, Giovanni Penco, Patrizia Bagnarelli, Valeria Micheli, Elisabetta Paolini, Antonio Di Biagio, Valeria Ghisetti, Massimo Di Pietro, Maurizio Zazzi, Andrea De Luca

**Affiliations:** 1 Infectious Diseases Clinic, Catholic University of the Sacred Heart, Rome, Italy; 2 Department of Pathology, Immunology and Laboratory Medicine, Emerging Pathogens Institute, College of Medicine, University of Florida, Gainesville, Florida, USA; 3 Department of Molecular Biology, University of Siena, Siena, Italy; 4 Microbiology and Virology Laboratory, San Martino Hospital, Genoa, Italy; 5 Department of Microbiology and Virology, Ospedali Riuniti di Bergamo, Bergamo, Italy; 6 Infectious Diseases Clinic, Ospedale Galliera, Genoa, Italy; 7 Virology Laboratory, Section of Microbiology, Department of Biomedical Sciences, Ospedale Generale di Torrette, Università Politecnica delle Marche, Ancona, Italy; 8 Microbiology Laboratory, L. Sacco Hospital, Milan, Italy; 9 Immuno-Ematology and Transfusion Medicine Service, Cremona Hospital, Cremona, Italy; 10 Infectious Diseases Clinic, San Martino Hospital, Genoa, Italy; 11 Virology Laboratory, Amedeo di Savoia Hospital, Turin, Italy; 12 Infectious Diseases Clinic, Santa Maria Annunziata Hospital, Florence, Italy; 13 Infectious Diseases Unit II, University Hospital of Siena, Siena, Italy

## Abstract

**Background:**

HIV-1 genotypic susceptibility scores (GSSs) were proven to be significant prognostic factors of fixed time-point virologic outcomes after combination antiretroviral therapy (cART) switch/initiation. However, their relative-hazard for the time to virologic failure has not been thoroughly investigated, and an expert system that is able to predict how long a new cART regimen will remain effective has never been designed.

**Methods:**

We analyzed patients of the Italian ARCA cohort starting a new cART from 1999 onwards either after virologic failure or as treatment-naïve. The time to virologic failure was the endpoint, from the 90^th ^day after treatment start, defined as the first HIV-1 RNA > 400 copies/ml, censoring at last available HIV-1 RNA before treatment discontinuation. We assessed the relative hazard/importance of GSSs according to distinct interpretation systems (Rega, ANRS and HIVdb) and other covariates by means of Cox regression and random survival forests (RSF). Prediction models were validated via the bootstrap and c-index measure.

**Results:**

The dataset included 2337 regimens from 2182 patients, of which 733 were previously treatment-naïve. We observed 1067 virologic failures over 2820 persons-years. Multivariable analysis revealed that low GSSs of cART were independently associated with the hazard of a virologic failure, along with several other covariates. Evaluation of predictive performance yielded a modest ability of the Cox regression to predict the virologic endpoint (c-index≈0.70), while RSF showed a better performance (c-index≈0.73, p < 0.0001 vs. Cox regression). Variable importance according to RSF was concordant with the Cox hazards.

**Conclusions:**

GSSs of cART and several other covariates were investigated using linear and non-linear survival analysis. RSF models are a promising approach for the development of a reliable system that predicts time to virologic failure better than Cox regression. Such models might represent a significant improvement over the current methods for monitoring and optimization of cART.

## Background

Modern combination antiretroviral therapy (cART) can suppress plasma viral load to undetectable levels in a large proportion of HIV-1 infected patients. The risk for a patient to experience virologic failure has been decreasing consistently during the last decade in high-income countries [1-5].

Part of this high rate of virologic success may be due to the increasing ability of more potent and tolerable antiretroviral drugs targeting a wider range of molecular targets, providing physicians the opportunity to tailor cART regimens according to patients' background and to manage treatment failure promptly. Additionally, better understanding of the mechanisms of drug resistance allows optimization of treatment regimens based on an individual's virus genotype. Although the prevalence of drug resistance seems to be decreasing in recent years [6], despite modern cART options [7], drug resistance remains a concern in chronically infected patients with a long treatment history and in treatment-naïve patients who have been infected with drug resistant isolates [8]. Overall, patients remain at risk of developing drug resistance.

Genotypic susceptibility scores (GSSs) have been developed for interpreting HIV-1 drug susceptibility based on the sequence of the virus genome coding for drug targets. GSSs usually consist of a set of general rules for scoring susceptibility to individual drugs. These systems are curated and updated by panels of experts and made freely available via the internet, and in some cases, as web-services. The most popular systems include the Stanford HIVdb [9], Agence Nationale de Recherche sur le Sida (ANRS) [10], and Rega [11] algorithms. Recently, machine learning methods have been also introduced to model HIV-1 drug resistance and to optimize cART design. These machine learning methods explore a larger set of variables besides the viral genotype (such as viral load, CD4+ T cell counts, demographic information, treatment history), and include techniques such as artificial neural networks [12], mutagenetic trees [13], Bayesian networks [14], and random forests [15]. Both GSSs and machine learning approaches have been proven to usefully predict virologic outcome at fixed time points (e.g. *n*-weeks) after cART initiation or switch [14-18]. The state-of-the art systems, available as free web-services, are able to select a set of suitable cARTs for a patient, given the patient's viral genotype and other background information, ensuring the maximal probability of viral load reduction after *n*-weeks of uninterrupted treatment [19,20].

Systems that predict the actual time to virologic failure, indicated by a viral load rebound following a cART-induced viral load reduction, or no viral load reduction after a few months of uninterrupted therapy, have not been developed yet. However, GSSs have been already associated with the time for achieving an undetectable viral load [18]. In this work we explore the predictive ability of linear and non-linear survival models with respect to the time to virologic failure endpoint, along with its prognostic factors. A model that could predict an individual's duration of virologic success with a cART regimen would provide valuable information in tailoring cART choice.

## Methods

### Study design

We considered patients enrolled in the Antiretroviral Resistance Cohort Analysis (ARCA), a national observational cohort [21] of HIV-1-infected patients followed up at > 100 clinical and laboratory units in Italy. At the time of this study, data from > 20,000 patients and > 23,000 HIV-1 *pol *gene sequences were available. Patients are anonymized and included in the ARCA database after signature of an informed consent to provide their data for academic not-for-profit studies. The ARCA initiative is compliant with the Declaration of Helsinki, and each participating centre is subject to a local ethics committee that follows national (and European where applicable) regulations.

Eligible patients were those starting a new cART from January 1999 onwards, comprising 2 nucleotide or nucleoside reverse transcriptase inhibitors (NRTI) plus either another NRTI or a non-nucleoside reverse transcriptase inhibitor (NNRTI), or a protease inhibitor (PI), or a ritonavir-boosted protease inhibitor (PI/r). Treatments included both a new therapy after virologic failure (defined as an HIV-1 RNA > 400 copies/ml while on therapy) and a first-line therapy in drug-naïve patients. Further selection criteria were a cART duration of more than 90 days, and the availability of at least one subsequent HIV-1 RNA determination after 90 days, using ultra-sensitive assays. Patients were excluded that had cART switches due to treatment simplification or early (e.g. < 90 days) changes of one or more drugs associated with tolerability/adherence issues.

The study endpoint was the time to virologic failure, quantified as the first HIV-1 RNA > 400 copies/ml beginning from the 90^th ^day after the cART start date and before the treatment discontinuation date. If the cART had not been discontinued yet, any HIV-1 RNA determination after the 90^th ^day of therapy was to be considered. Data were censored at the last available HIV-1 RNA determination < = 400 copies/ml available before the treatment stop date, ignoring HIV-1 RNA determinations after the cART discontinuation.

The following variables were coupled with each patient's record of cART switch/initiation and subsequent HIV-1 RNA determinations: calendar year; baseline HIV-1 RNA, obtained within [-90, 0] days from the cART start date, without other treatment changes during that time interval; baseline CD4+ T cell count, [-90, +30] days from the cART start date; age; gender; mode of HIV-1 transmission; nationality; previous AIDS-defining events, hepatitis B/C virus co-infection (either HBsAg or HCV antibody positive serostatus); time passed from the first HIV-1 positive antibody test to the first cART initiation; duration of prior antiretroviral exposure; number of previous antiretroviral therapy switches (any drug change for any reason); previous drug class exposures (combinations of NRTI/NNRTI/PI/other classes); previous suboptimal treatment (less than three drugs in a regimen); achievement of an HIV-1 RNA < = 50 copies/ml at any time point during the cART follow up; a baseline HIV-1 *pol *genotype (encompassing at least positions 1-99 in the protease and positions 1-250 in the reverse transcriptase), obtained within [-90, +15] days from the cART start date, without other therapies used during that time interval except the failing regimen, when applicable.

The baseline HIV-1 genotype was successively processed by calculating the GSS using the latest available version from 3 interpretation systems (Rega 8.0.2, ANRS 2009.07, and HIVdb 6.0.9) with respect to the associated cART. We used the standard susceptible/intermediate/resistant categorization for all GSS, as by the output by the HIVdb web-service [9], which were assigned the numerical values of 1.0/0.5/0.0, respectively. The algebraic sum of GSS, calculated for drugs included in the cART coupled to each genotype, was regarded as the overall GSS of that cART regimen. Viral subtype was determined with the Rega subtyping tool [22]. Unassigned subtypes were defined as undetermined.

### Statistical analysis

Cox multivariable proportional hazard regression (with robust variance estimation via a grouped jackknife) [23] and random survival forests (RSF) [24] were considered (with 30 to 100 single trees to be grown).

The Cox proportional hazard model is a general non-parametric regression technique which is not based on any assumptions concerning the underlying survival distribution. The model assumes that the underlying hazard rate (rather than survival time) is a function of the independent covariates. Let *Y_i _*denote the observed time (either censoring time or event time) for subject *i*, and let *C_i _*be the indicator that the time corresponds to an event (i.e. if *C_i _*= 1 the event occurred and if *C_i _*= 0 the time is a censoring time). The hazard function Λ(*t|X*) for the Cox proportional hazard model has the form

This expression gives the hazard at time *t *for an individual with covariate vector (explanatory variables) *X*. The term Λ_0_(*t*) is called the baseline hazard, and it is the hazard for the respective individual when all independent variable values are equal to zero. The model is called proportional hazard because, while no assumptions are made about the shape of the underlying hazard function, the model equations specify a multiplicative relationship between the underlying hazard function and the log-linear function of the covariates. There is a log-linear relationship between the independent variables and the underlying hazard function. In addition, given two observations with different values for the independent variables, the ratio of the hazard functions for those two observations does not depend on time.

RSF are an extension used to analyze censored data of the random forests machine-learning method, an ensemble of several decision trees for classification and regression. A random survival tree is a special form of decision tree for survival analysis. The tree is constructed using a training data set made of observation data points of time, status/event, and associated predictor covariates. A binary tree is grown by inferring node splits upon the set of covariates as follows. The space of observations is recursively divided into two disjoint sub-spaces, thus inferring a node split, based on an optimal cut-off value of a predictor. The log-rank statistic is used usually as a criterion for node splitting in survival trees. In detail, a proposed split at node *h *on a given predictor *x *takes always the form *x *≤ *c *and *x *>*c*. This split induces two children nodes and two sub-sets of survival data. A good split should maximize survival differences across the two sets of data. Let *t_1 _*<*t_2 _*< ... <*t_n _*be the distinct death times in the parent node *h*, and let *d_i,j _*and *Y_i,j _*equal the number of deaths and individuals at risk at time *t_i _*in the children nodes *j *= 1, 2. Note that *Y_i,j _*is the number of individuals in the child node *j *who are alive at time *t_i_*, or who have an event (death) at time *t_i_*. The log-rank statistics for a node split at value *c *for a variable *x *is

The larger the absolute value for *L (x,c) *is, the better the split is. Each tree node contains the number of total and censored observations falling into the current category, as well as a Kaplan-Maier estimation of the cumulative survival for the group is calculated at the end nodes. Since the predictive performance of one survival/decision tree can be poor, different trees can be combined together to obtain improved performance. RSF are an ensemble average of different survival trees. Each tree is grown on different bootstrap samples of the original data set, and a randomization is introduced in the recursive node splitting phase by considering a random sub-set of predictors at each step. These characteristics enable to approximate complex functions with a generally low generalization error. One theoretical advantage of RSF over the Cox regression is that the latter relies on the restrictive assumption of the proportional hazards. In addition, RSF manage automatically the non-linear interactions among variables, whilst in Cox regression non-linear and higher-order interactions need to be explored explicitly.

Cox regression and RSF models were fitted on the whole study population and on the subset of therapy-naïve patients. An additional sensitivity analysis was carried out by considering only those patients with at least two follow-up HIV-1 RNA determinations, where a HIV-1 RNA viral load < = 50 copies/ml occurred at any time point during the cART follow up.

The predictive ability of the RSF and Cox regression was evaluated by means of the Harrell's c-index measure [25], comparing either linear predictions of Cox regression or mortality rates of RSF against observed time/event pairs, using the bootstrap .632 method (100 runs) for assessing the generalization error on unseen data [26]. The c-index is defined as the probability of *agreement *for any two randomly chosen observations, where agreement means that the observation with the shorter survival time of the two also has the larger risk score. A previous study in the different context of breast cancer research successfully used the c-index to compare RFS and Cox regression [27].

The free software environment for statistical computing and graphics "R" was employed for all statistical analyses and graph generations [28]. Besides the "base" package, the "survival", "randomSurvivalForest", and "Hmisc" libraries were used to fit Cox regression models, RSF and to calculate c-index statistics, respectively.

## Results

A total of 2,337 cART regimens from 2,158 patients were considered. The proportion of patients who were previously therapy-naïve was 34% (n = 733). Table 1 summarizes patients' characteristics.

**Table 1:** Characteristics of the study population

*Numerical variables*		*all patients*	*ART-naive*	*ART-experienced*
		***median (IQR)***	***median (IQR)***	***median (IQR)***

calendar year		2004 (2003-2006)	2006 (2004-2007)	2004 (2002-2005)

HIV-1 RNA log10 copies/ml		4.53 (3.83-5.12)	5.08 (4.63-5.5)	4.18 (3.63-4.8)

CD4+ count cells/mm3		273 (147-389)	191 (70-300)	296.8 (192-435)

Age years		40 (36-45)	39 (33-45)	40 (37-45)

GSS	Rega	3 (2-3)	3 (3-3)	2 (1.5-3)
	
	ANRS	3 (2-3)	3 (3-3)	2 (1.5-3)
	
	HIVdb	3 (2-3)	3 (3-3)	2 (1.5-3)

*Categorical variables*		*n (%)*	*n (%)*	*n (%)*

#patients		2158	733 (34.0%)	1425 (57.7%)

#cART regimens		2337	733 (31.4%)	1604 (68.6%)

cART type	2NRTI+1NNRTI	516 (23.9%)	235 (32.1%)	281 (19.7%)
	
	2NRTI+1PI	243 (11.3%)	45 (6.1%)	198 (13.9%)
	
	2NRTI+1PI/r	1306 (60.5%)	441 (60.2%)	865 (60.7%)
	
	3NRTI	93 (4.3%)	12 (1.6%)	81 (5.7%)

gender	male	1546 (71.6%)	563 (76.8%)	983 (69%)

previous AIDS-defining events (yes vs. no)		285 (13.2%)	92 (12.6%)	193 (13.5%)

Nationality	Italian	1524 (70.6%)	529 (72.2%)	995 (69.8%)
	
	non-Italian	168 (7.8%)	98 (13.4%)	70 (4.9%)
	
	unknown	466 (21.6%)	106 (14.5%)	360 (25.3%)

mode of HIV-1 transmission	heterosexual	657 (30.4%)	262 (35.7%)	395 (27.7%)
	
	male homosexual	362 (16.8%)	154 (21%)	208 (14.6%)
	
	IDU	468 (21.7%)	54 (7.4%)	414 (29.1%)
	
	other/unknown	671 (31.1%)	263 (35.9%)	408 (28.6%)

HBV/HCV co-infection	positive	605 (28%)	149 (20.3%)	456 (32%)
	
	negative	483 (22.4%)	260 (35.5%)	223 (15.6%)
	
	unknown	1070 (49.6%)	324 (44.2%)	746 (52.4%)

interval time from the first HIV-1 positive test to cART initiation	< = 12 months	444 (20.6%)	354 (48.3%)	90 (6.3%)
	
	> 12 and < = 60 months	188 (8.7%)	45 (6.1%)	143 (10%)
	
	> 60 months	575 (26.6%)	61 (8.3%)	514 (36.1%)
	
	unknown	951 (44.1%)	273 (37.2%)	678 (47.6%)

duration of prior ART exposures	< = 6 months	1197 (55.5%)	733 (100%)	464 (32.6%)
	
	> 6 and < = 12 months	117 (5.4%)	N/A	117 (8.2%)
	
	> 12 and < = 24 months	184 (8.5%)	N/A	184 (12.9%)
	
	> 24 months	660 (30.6%)	N/A	660 (46.3%)

viral subtype	B	1753 (81.2%)	503 (68.6%)	1250 (87.7%)
	
	02_AG	56 (2.6%)	28 (3.8%)	28 (2%)
	
	C	41 (1.9%)	31 (4.2%)	10 (0.7%)
	
	F1	54 (2.5%)	39 (5.3%)	15 (1.1%)
	
	other	60 (2.8%)	35 (4.8%)	25 (1.8%)
	
	undetermined	194 (9%)	97 (13.2%)	97 (6.8%)

#previous ART switches	none	733 (34%)	733 (100%)	N/A
	
	one/two	429 (19.9%)	N/A	429 (30.1%)
	
	three to six	605 (28%)	N/A	605 (42.5%)
	
	more than six	391 (18.1%)	N/A	391 (27.4%)

previous exposure to suboptimal ART (yes vs. no)		922 (42.7%)	N/A	922 (64.7%)

previous ART class exposure	none	733 (34%)	733 (100%)	N/A
	
	only NRTI	94 (4.4%)	N/A	94 (6.6%)
	
	NRTI and NNRTI	220 (10.2%)	N/A	220 (15.4%)
	
	NRTI, NNRTI and PI	252 (11.7%)	N/A	252 (17.7%)
	
	NRTI, NNRTI and PI/r	466 (21.6%)	N/A	466 (32.7%)
	
	NRTI and PI	229 (10.6%)	N/A	229 (16.1%)
	
	NRTI and PI/r	134 (6.2%)	N/A	134 (9.4%)
	
	other classes	30 (1.4%)	N/A	30 (2.1%)

*cART: combination antiretroviral therapy; GSS: genotypic susceptibility score; ART: antiretroviral therapy; NRTI: nucleoside/tide reverse transcriptase inhibitors; NNRTI: non-nucloeside reverse transcriptase inhibitors; PI: protease inhibitors; IDU: injecting drug users; HCV: hepatitis C virus; HBV: hepatitis B virus.

We observed 1,067 virologic failures over 2,820 person years of follow-up (rate = 37.8 per 100 person years). By Kaplan-Meier estimation, median (95% CI) time to virologic failure was 659 (533-784) days for all patients, and 2,510 (1,715-N/A) days for those previously therapy-naïve. By two years, the estimated proportion of patients not experiencing virologic failure was 0.48 (0.46-0.51) when considering the whole set of patients, and 0.71 (0.67-0.75) for those previously therapy-naïve.

Multivariable Cox analysis revealed that higher GSSs (each fitted in separate models including the other covariates), a more recent calendar year, patients administered 2NRTI+1PI/r, as compared to those undertaking 2NRTI+1NNRTI, and younger age were independently associated with a decreased hazard of virologic failure. Conversely, a higher HIV-1 RNA, a lower CD4+ count, and previous drug class exposure were associated with an increased hazard. Table 2 summarizes the results. Variable importance and partial standardized mortality plots of RSF (fitted using 100 trees) in general confirmed the Cox hazards (see Additional file 1, supplementary figures S1 and S2).

**Table 2:** Multivariable Cox proportional hazard model showing relative hazards (RH) for time-to-virologic-failure, fitted on the whole study population (n = 2,337)

*Factor*		*RH*	*95% CI*	*p-value*
calendar year	before 2004 vs. 2007 and after	2.06	(1.67-2.54)	< 0.0001
	
	2004 vs. after 2007 and after	1.62	(1.29-2.03)	< 0.0001
	
	2005-2006 vs. 2007 and after	1.28	(1.06-1.55)	0.0109

cART	2NRTI+1PI vs. 2NRTI+1NNRTI	1.03	(0.83-1.27)	0.8028
	
	2NRTI+1PI/r vs. 2NRTI+1NNRTI	0.63	(0.54-0.75)	< 0.0001
	
	3NRTI vs. 2NRTI+1NNRTI	1.23	(0.92-1.64)	0.1599

age (per 10 years older)		0.89	(0.82-0.96)	0.0036

gender (male vs. female)		1.06	(0.91-1.23)	0.4668

mode of HIV-1 transmission	male homosexual vs. heterosexual	1.08	(0.88-1.33)	0.4680
	
	IDU vs. heterosexual	1.08	(0.87-1.32)	0.4898
	
	other/unknown vs. heterosexual	1.08	(0.9-1.29)	0.4248

nationality	non-Italian vs. Italian	1.23	(0.9-1.67)	0.1992
	
	unknown vs. Italian	0.94	(0.78-1.14)	0.5475

HCV/HBV coinfection	unknown vs. no	1.18	(0.97-1.45)	0.1049
	
	yes vs. no	1.04	(0.82-1.32)	0.7277

HIV-1 RNA per log10 copies/ml higher		1.27	(1.17-1.39)	< 0.0001

CD4+ count cells/mm3	< = 100 vs. > 500	1.57	(1.23-2)	0.0003
	
	> 100 and < = 199 vs. > 500	1.16	(0.93-1.45)	0.1968
	
	> 200 and < = 349 vs. > 500	1.22	(1-1.48)	0.0447
	
	> 350 and < = 499 vs. > 500	0.98	(0.79-1.21)	0.8202

interval time from the first HIV-1 positive test to ART initiation	< = 12 vs. > 60 months	0.87	(0.67-1.13)	0.2944
	
	> 12 and < = 60 vs. > 60 months	1.01	(0.81-1.27)	0.9114
	
	unknown vs. > 60 months	0.92	(0.77-1.11)	0.3938

duration of prior ART exposures	< = 6 vs. > 24 months	0.84	(0.7-1.01)	0.0626
	
	> 6 and < = 12 vs. > 24 months	0.92	(0.73-1.17)	0.5029
	
	> 12 and < = 24 vs. > 24 months	0.83	(0.66-1.03)	0.0890

previous AIDS-defining events (yes vs. no)		0.86	(0.7-1.05)	0.1379

#previous ART switches		1.03	(1-1.05)	0.0522

previous ART class exposure	NRTI vs. ART-naïve	1.48	(1.01-2.17)	0.0441
	
	NRTI and NNRTI vs. ART-naïve	1.38	(0.99-1.93)	0.0546
	
	NRTI and NNRTI and PI vs. ART-naïve	1.43	(1.03-1.99)	0.0315
	
	NRTI and NNRTI and PI/r vs. ART-naïve	2.96	(2.16-4.06)	< 0.0001
	
	NRTI and PI vs. ART-naïve	2.18	(1.64-2.89)	< 0.0001
	
	NRTI and PI/r vs. ART-naïve	2.72	(1.98-3.75)	< 0.0001
	
	other classes vs. ART-naïve	2.31	(1.31-4.05)	0.0036

previous exposure to suboptimal ART (yes vs. no)		0.85	(0.71-1.03)	0.0946

viral subtype	02_AG vs. B	0.98	(0.61-1.57)	0.9402
	
	C vs. B	1.41	(0.86-2.32)	0.1748
	
	F1 vs. B	0.57	(0.3-1.06)	0.0750
	
	other vs. B	1.26	(0.76-2.09)	0.3774
	
	undetermined vs. B	1.12	(0.88-1.42)	0.3527

GSS*	ANRS per 1 point increase	0.72	(0.66-0.78)	< 0.0001
	
	HIVdb per 1 point increase	0.68	(0.63-0.74)	< 0.0001
	
	Rega per 1 point increase	0.71	(0.66-0.77)	< 0.0001

RH: relative hazard; CI: confidence interval; cART: combination antiretroviral therapy; NRTI: nucleoside/nucleotide reverse transcriptase inhibitors; NNRTI: non-nucloeside reverse transcriptase inhibitors; PI: protease inhibitors; PI/r: ritonavir-boosted PI; IDU: injecting drug users; HCV: hepatitis C virus; HBV: hepatitis B virus; ART: antiretroviral therapy; GSS: genotypic susceptibility score; *fitted separately one from each other.

**Figure 1:**
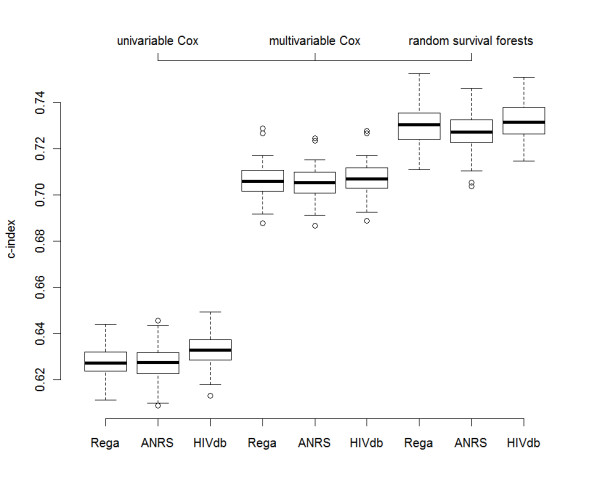
**Extra-sample performance evaluation of Cox regression and Random Survival Forests models by means of c-index distributions (100 bootstrap runs)**. Boxplots indicate average and interquartile range, whilst whiskers indicate 95% confidence intervals.

The evaluation of extra-sample predictive performance (via the bootstrap .632 method, on 100 runs) is shown in Figure 1. In detail, the multivariable Cox models fitted with different GSSs yielded an average (st.dev) c-index of 0.7060 (0.007) for Rega, 0.7048 (0.007) for ANRS, and 0.7068 (0.007) for HIVdb. As expected, univariable Cox models fitted with the single GSSs were greatly outperformed by their multivariable versions, yielding -respectively- an average (st.dev) c-index of 0.6277 (0.007), 0.6271 (0.007), and 0.6330 (0.007). Additionally, a likelihood-ratio-test conducted on the Cox regression, considering a null model with the sole GSS and an extended model with all covariates, reported a consistently worse fit of the null model as compared to the extended model (L_null _= -7573.536, L_extended _= -7361.47, χ^2 ^= 424.13 on 41 degrees of freedom, p < 0.0001).

The c-index performance of RSF, grown with the limited number of 30 trees due to the high computational burden, using the same covariate settings as in the multivariable Cox regression, were 0.7298 (0.009) for Rega, 0.7276 (0.009) for ANRS, and 0.7319 (0.008) for HIVdb.

By executing all pairwise Student's t-tests (adjusted for multiple comparisons with the Benjamini-Hochberg method) comparing the c-index distributions, we find statistically significant differences between univariable vs. multivariable Cox models, univariable Cox models vs. RSF, and multivariable Cox models vs. RSF (all p < 0.0001). When comparing the performance of Rega, ANRS and HIVdb within the same model settings, we found evidence of a significantly better performance of HIVdb as compared to ANRS in the univariable Cox (p < 0.0001), multivariable Cox and (p = 0.053), and RSF (p = 0.0005). HIVdb outperformed Rega only under the univariable Cox modeling. Conversely, Rega and ANRS did not show any appreciable difference in any of the within-model c-index distributions. However, it has to be noted that all the GSS had the same average c-index values up to the third decimal.

When executing a sensitivity analysis on the subset of treatment-naïve patients (n = 733), multivariable Cox regression confirmed the relative hazards of the GSS of the regimen (RH = 0.50, 95%CI 0.36-0.70, p < 0.0001, per one point increase of HIVdb), and of HIV-1 RNA (RH = 1.51, 95%CI 1.15-1.97, p = 0.0026, per one Log10 copies/ml higher). Other factors independently associated with the endpoint were being non-Italian born (RH = 1.87, 95%CI 1.09-3.22, p = 0.023, as compared to Italian born), subtype C (RH = 2.16, 95%CI 1.05-4.44, p = 0.036, as compared to subtype B), and calendar year 2004 (RH = 2.08, 95%CI 1.31-3.31, p = 0.0019, as compared to 2007 and after). Differences among cART regimens were not relevant.

In a second sensitivity analysis, only patients who reached an HIV-1 RNA < = 50 copies/ml from the cART start date onwards were selected (n = 1,578, of which 622 treatment-naïve). By fitting a multivariable Cox regression, variables independently associated with the endpoint were consistent with those obtained for the subset of treatment-naïve patients (data not shown).

## Discussion

In this study we investigated linear and non-linear survival models for predicting the time to virologic failure in HIV-1-infected patients undergoing a new cART regimen, with the aim to assess both prognostic factors of virologic failure and performance of predictions, in light of the development of a reliable expert system.

In contrast to 2NRTI+1PI/r, older age, higher HIV-1 RNA, and lower CD4+ counts, an increased hazard of virologic failure was associated with low GSSs of cART, a less recent calendar year, administration of 2NRTI+1NNRTI, and the ART-naïve status. HIV-1 RNA and GSS remained associated with the endpoint when considering only treatment-naïve patients in a sensitivity analysis.

When looking at the goodness-of-fit of the models, the inclusion of additional covariates besides the GSS in a multivariable Cox regression yielded a significant improvement in the likelihood. Furthermore, RSF proved to be a promising approach, improving performance over that obtained by using the Cox method.

This study has some limitations. First, there was a study selection bias, since only cART regimens that were undertaken for at least 90 days were considered. Early-switches and simplifications were excluded from the analysis. In addition, the main study endpoint was a pure virologic criterion and did not include stops due to other reasons. Although we selected only clinics with the ability to perform ultra-sensitive assays, the viral load threshold at > 400 copies/ml was arbitrary and might not capture all the actual virologic failures. Other thresholds for defining virologic failure, such as a viral load > 50 copies/ml or > 1,000 copies/ml might overestimate or underestimate true failure, respectively. We observed a lower hazard of virologic failure for regimens containing ritonavir-boosted PI as compared to those containing NNRTI, and this might be a reflection of both the selection bias and the endpoint definition.

In regards to the statistical methods, we did not investigate the potential benefit in terms of likelihood fit given by the inclusion of interaction terms in the Cox regression. It is possible that a Cox model with higher-order interactions is able to reach the performance obtained by using the RSF. For the practical perspective of a time to event prediction model, Cox regression presents some problems with the baseline hazard function estimation, while the RSF gives output in terms of mortality ensembles. We also tested accelerated failure time models, which are able to give reliable predictions in terms of actual time scales, and their performance was comparable to the results of the Cox regression (data not shown).

## Conclusions

This study demonstrates the feasibility of an expert system that predicts the actual time course of a new cART as the estimate of time to virologic failure, and the reliability of the system's predictions can be improved both by including additional covariates besides the viral genotype and by using non-linear regression techniques. The implementation of such a system would create a more clinically-oriented treatment decision tool and more effectively tailor patient cART regimens.

## Competing interests

ADL received speakers' honoraria, served as consultant or participated in advisory boards for GlaxoSmithKline, Gilead, Bristol-Myers Squibb, Abbott Virology, Tibotec-Janssen, Siemens Diagnostics, and Monogram Biosciences.

MZ received research funding from Pfizer, served as a consultant for Abbott Molecular, Boehringer Ingelheim, Gilead Sciences, and Janssen, and served on speakers' bureaus for Abbott, Bristol-Myers Squibb, Merck, and Pfizer.

SDG received speakers' honoraria or consultancy fees from Abbott, GlaxoSmithKline, Gilead, Boehringer Ingelheim, Jansen-Tibotec, Merck-Sharp & Dohme, and Bristol Myers Squibb.

BB has received funds for speaking, consultancy and travel from ViiV Healthcare, Gilead Sciences, Abbott Molecular, Janssen-Cilag and Siemens Health Care.

Other authors: none to declare.

## Authors' contributions

MCFP: statistical analyses, machine learning modeling, manuscript writing; SDG: study design; IF: data base administration, data extraction; GM, BB, AC, GP, PB, VM, EP, ADB, VG, MDP: local data base administration, local data manipulation and provision to the ARCA cohort, clinical activity, patient care; MZ: molecular biology expertise, sequencing; ADL: research group leading, manuscript revision. All authors read and approved the final manuscript.

## Pre-publication history

The pre-publication history for this paper can be accessed here:

http://www.biomedcentral.com/1472-6947/11/40/prepub

## Supplementary Material

Additional file 1 **Supplementary material**. Supplementary figures describing performance and variable importance measures of Random Survival Forests. Click here for file
