# Supplementary material for: A Prognostic Model for Estimating the Time to Virologic Failure in HIV-1 Infected Patients Undergoing a New Combination Antiretroviral Therapy Regimen
Source: BMC Med Inform Decis Mak. 2011 Jun 14;11:40. doi: 10.1186/1472-6947-11-40 (PMC3144446; doi:10.1186/1472-6947-11-40)

**Additional file 1**

**Supplementary figure S1.** Error rate assessment (1-c_index, out of bag) by tree growth in the random survival forests and variable importance evaluation (full data set, n=2,337). Large importance values indicate variables with predictive ability, whereas zero or negative values identify non-predictive variables to be filtered.


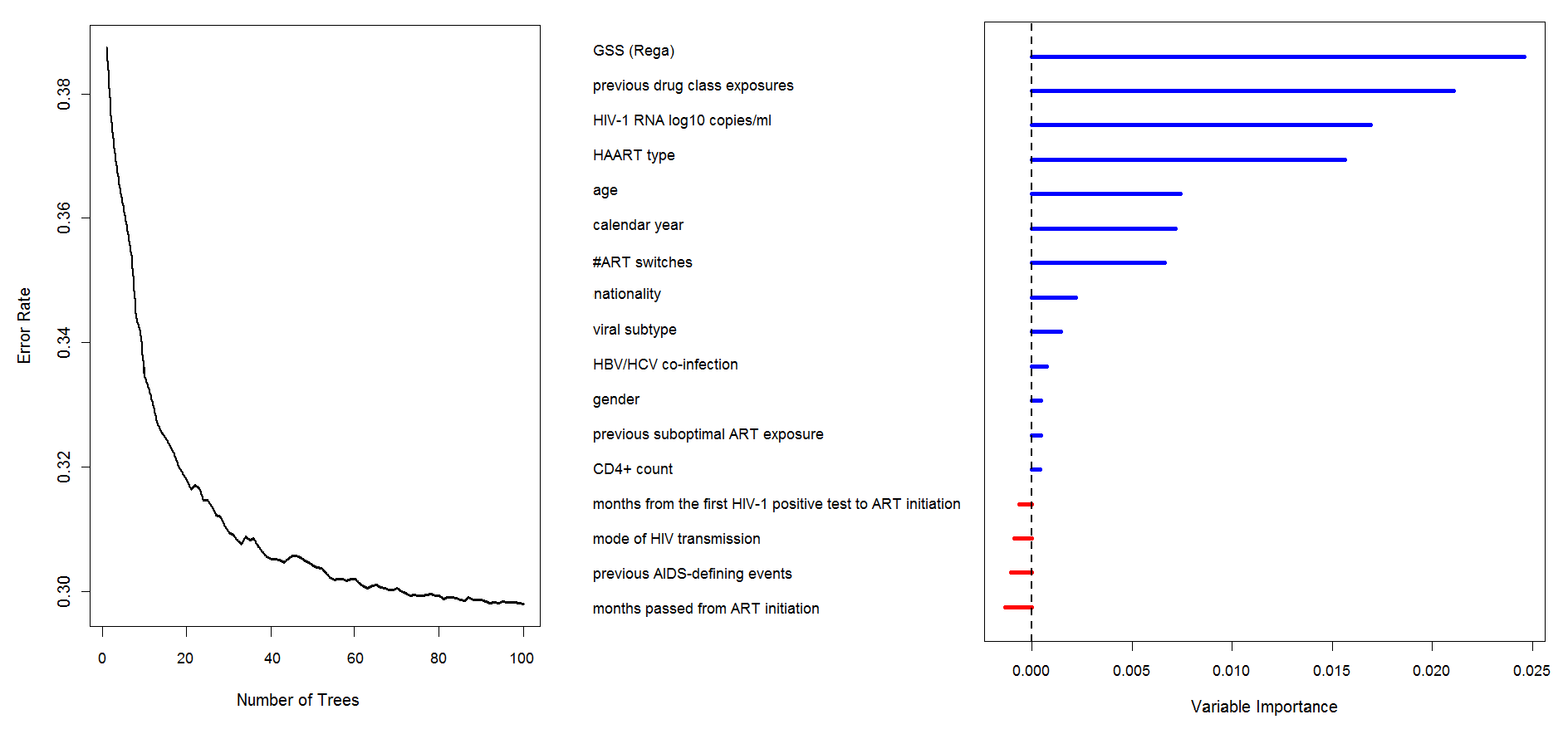


**Supplementary figure S2.** Partial standardized mortality plots by means of a random survival forests model (full data set, n=2,337).


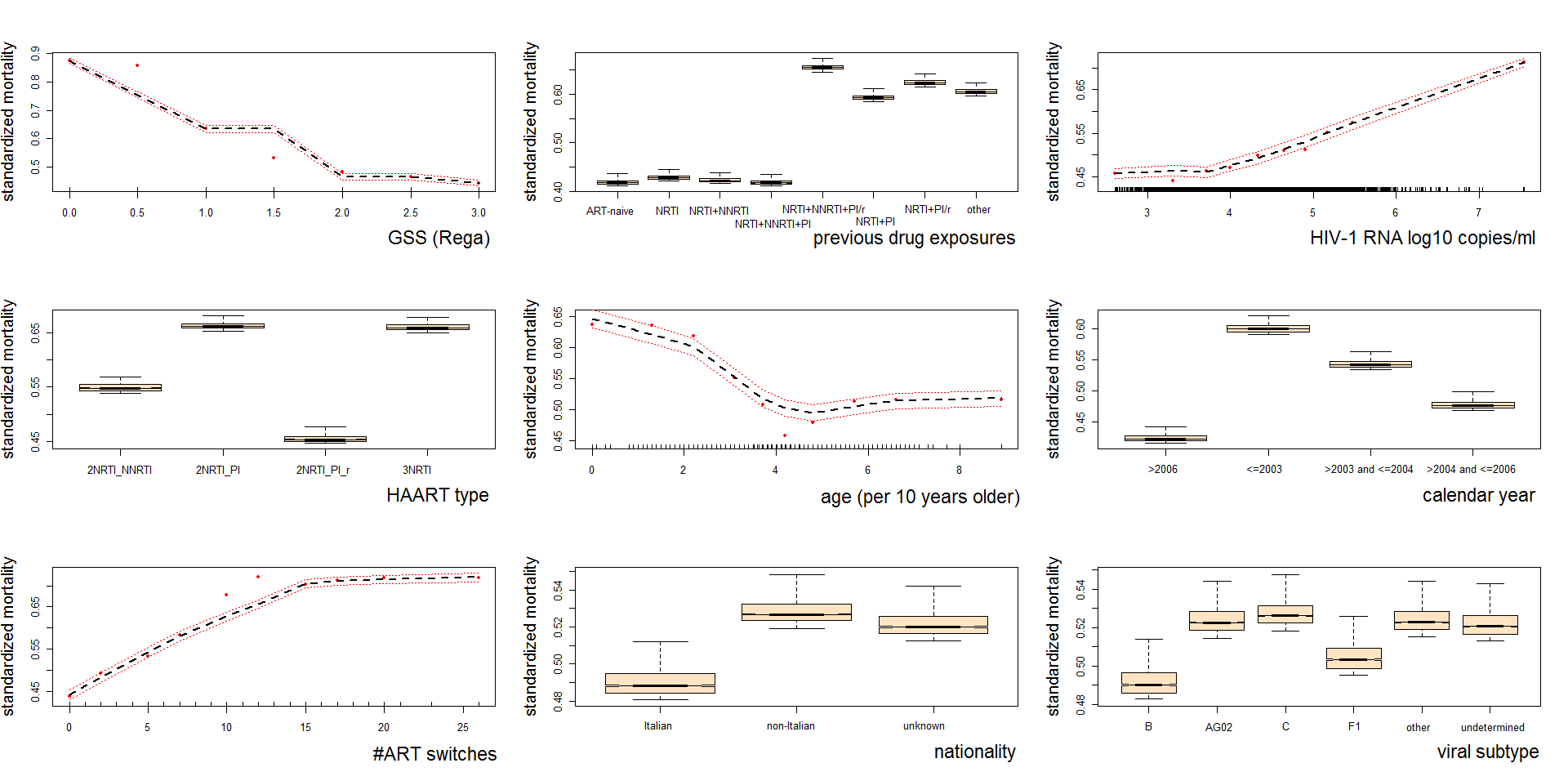

Supplement: Additional file 1 — Supplementary material. Supplementary figures describing performance and variable importance measures of Random Survival Forests. [file 1472-6947-11-40-S1.DOC]
